# Mechanisms and Treatment of Light-Induced Retinal Degeneration-Associated Inflammation: Insights from Biochemical Profiling of the Aqueous Humor

**DOI:** 10.3390/ijms21030704

**Published:** 2020-01-21

**Authors:** Dmitry V. Chistyakov, Viktoriia E. Baksheeva, Veronika V. Tiulina, Sergei V. Goriainov, Nadezhda V. Azbukina, Olga S. Gancharova, Eugene A. Arifulin, Sergey V. Komarov, Viktor V. Chistyakov, Natalia K. Tikhomirova, Andrey A. Zamyatnin, Pavel P. Philippov, Ivan I. Senin, Marina G. Sergeeva, Evgeni Yu. Zernii

**Affiliations:** 1 Belozersky Institute of Physico-Chemical Biology, Lomonosov Moscow State University, 119992 Moscow, Russia; vbaksheeva@belozersky.msu.ru (V.E.B.); tyulina_nika@list.ru (V.V.T.); olgancharova@belozersky.msu.ru (O.S.G.); tikhomir@belozersky.msu.ru (N.K.T.); ppph@belozersky.msu.ru (P.P.P.); senin@belozersky.msu.ru (I.I.S.); mg.sergeeva@gmail.com (M.G.S.); 2 Skryabin Moscow State Academy of Veterinary Medicine and Biotechnology, 109472 Moscow, Russia; skomarov1977@mail.ru; 3 SREC PFUR Peoples’ Friendship University of Russia (RUDN University), 117198 Moscow Russia; goryainovs@list.ru (S.V.G.); chistvic@gmail.com (V.V.C.); 4 Faculty of Bioengineering and Bioinformatics, Moscow Lomonosov State University, 119234 Moscow, Russia; ridernadya@gmail.com; 5 Institute of Molecular Medicine, Sechenov First Moscow State Medical University, 119991 Moscow, Russia

**Keywords:** ocular inflammation, light-induced retinal damage, age-related macular degeneration, oxidative stress, polyunsaturated fatty acids, oxylipins, mitochondria-targeted antioxidant, SkQ1, non-steroidal anti-inflammatory drugs, Nepafenac

## Abstract

Ocular inflammation contributes to the pathogenesis of blind-causing retinal degenerative diseases, such as age-related macular degeneration (AMD) or photic maculopathy. Here, we report on inflammatory mechanisms that are associated with retinal degeneration induced by bright visible light, which were revealed while using a rabbit model. Histologically and electrophysiologically noticeable degeneration of the retina is preceded and accompanied by oxidative stress and inflammation, as evidenced by granulocyte infiltration and edema in this tissue, as well as the upregulation of total protein, pro-inflammatory cytokines, and oxidative stress markers in aqueous humor (AH). Consistently, quantitative lipidomic studies of AH elucidated increase in the concentration of arachidonic (AA) and docosahexaenoic (DHA) acids and lyso-platelet activating factor (lyso-PAF), together with pronounced oxidative and inflammatory alterations in content of lipid mediators oxylipins. These alterations include long-term elevation of prostaglandins, which are synthesized from AA via cyclooxygenase-dependent pathways, as well as a short burst of linoleic acid derivatives that can be produced by both enzymatic and non-enzymatic free radical-dependent mechanisms. The upregulation of all oxylipins is inhibited by the premedication of the eyes while using mitochondria-targeted antioxidant SkQ1, whereas the accumulation of prostaglandins and lyso-PAF can be specifically suppressed by topical treatment with cyclooxygenase inhibitor Nepafenac. Interestingly, the most prominent antioxidant and anti-inflammatory benefits and overall retinal protective effects are achieved by simultaneous administrating of both drugs indicating their synergistic action. Taken together, these findings provide a rationale for using a combination of mitochondria-targeted antioxidant and cyclooxygenase inhibitor for the treatment of inflammatory components of retinal degenerative diseases.

## 1. Introduction

Inflammation is implicated in the etiology and progression of a number of blind-causing retinal degenerative diseases. Normally, the retina represents an immune-privileged zone that is separated by the inner and outer blood-retinal barriers that formed by retinal pigment epithelium (RPE) cells and microvascular endothelial cells, respectively [1,2]. However, retinal degenerative conditions associated with environmental, age-related, vascular, metabolic, and/or genetic factors may deteriorate these barriers and, thereby, contribute to the propagation of intraocular inflammation affecting the retina [1]. Oxidative stress plays a critical role in triggering retinal degeneration and intraocular inflammation. Photoreceptor and RPE cells generate high levels of reactive oxygen species (ROS) due to constant exposure to light and the presence of multiple photosensitizer molecules. In addition, extremely high oxygen consumption and metabolic rates characterize the retina, which underlie its high susceptibility to mitochondrial oxidative stress [1,3].

Oxidative stress and inflammation contribute to the development of age-related macular degeneration (AMD), diabetic retinopathy, retinopathy of prematurity, and retinitis pigmentosa [1,4]. For instance, pathological extracellular deposits (the so-called drusen) that are found in the retina of AMD patients contain byproducts of active inflammation and complement activation, together with products of lipid peroxidation [5,6]. Intraocular inflammation and macular edema can also be of iatrogenic origin, representing a complication of intraocular surgery and laser procedures. Without treatment, these complications can promote generalized breakdown of the blood-retinal barrier and irreversible retinal damage [7]. Light-induced retinal degeneration (LIRD) is a major driving force of photic maculopathy (light maculopathy, light-induced retinopathy), another common condition that shares many features with the above-mentioned ocular diseases [8]. Being conveniently simulated in animals, LIRD models are commonly employed for studies of pathogenesis and trailing of therapeutic approaches with respect to these diseases [9]. Spontaneous photochemical damage to the retina can be induced, for instance, by intensive light exposure from ophthalmological illuminators [10,11,12]. As an aggravating factor, excessive light illumination of the retina contributes to AMD and other retinal degenerative disorders by inducing the apoptosis of photoreceptors and other pathological hallmarks [8,13]. LIRD also resembles retinal degenerative diseases in terms of the pathogenetic roles of oxidative stress and inflammation. Prolonged illumination of the retina with visible light induces photochemical reactions in photoreceptors and RPE cells, yielding an accumulation of ROS and the oxidation of lipids and proteins [3,14,15] characteristic to AMD and other degenerative retinopathies [8,16]. Furthermore, LIRD might induce breakage of the blood-retinal barrier thereby promoting leukocyte infiltration and macular edema [14,17], and these signs are also can be found in AMD patients [18,19].

The exact mechanisms underlying induction and resolution of inflammation associated with degenerative retinal diseases remain mostly unspecified. The development of inflammation in AMD, diabetic retinopathy, retinitis pigmentosa, retinopathy of prematurity, and glaucoma seems to be regulated by interleukin-1 (IL-1) family members, as well as tumor necrosis factor alpha (TNF-α) and its counterpart interleukin-10 (IL-10) [20,21,22]. Consistently, the expression levels of interleukin-1 beta (IL-1β) and TNF-α were found to be altered in animal models of LIRD [23,24]. There are evidences that polyunsaturated fatty acids (PUFAs)-derived lipid mediators, such as oxylipins and resolvins, control the inflammatory component of retinal degenerative diseases. For instance, arachidonic acid (AA)-derived oxylipins, prostaglandins (PGs), were shown to participate in the pathogenesis of AMD and diabetic retinopathy [25]. Generally, the biosynthesis of oxylipins occurs via enzymatic pathways that involve cyclooxygenases (COX), lipoxygenases (LOX), or cytochrome P450 monooxygenases (CYP), and prostaglandins are generated in a COX-dependent pathway [26]. Consistently, nonsteroidal anti-inflammatory drugs (NSAIDs) that represent COX inhibitors were suggested for the treatment of AMD, diabetic retinopathy, and macular edema [27]. The increased expression levels of retinal COX-2 was also observed in LIRD, pointing on similar mechanisms of triggering inflammation [23,24]. Notably, certain oxylipins are synthesized via the non-enzymatic pathway due to the oxidation of PUFAs by free radicals. The highest content of PUFAs, particularly DHA and AA, characterize the retina and, therefore, it is extremely vulnerable to lipid peroxidation yielding corresponding specific oxylipins [16,28]. For instance, LIRD is associated with a pronounced elevation in the retinal concentration of oxylipin 8-iso-prostaglandin F2 alpha (8-iso-PGF_2α_), representing a product of free radical oxidation of AA independent of COX pathways [29,30]. In this case, the retina was also found to contain high amounts of hydroxyoctadecadienoic acids (HODEs), linoleic acid (LA)-derived oxylipins, which can be biosynthesized by enzymatic and non-enzymatic pathways [29]. Thus, oxylipins can be regarded as appropriate biomarkers that respond to both retinal inflammation and oxidative stress.

Intraocular inflammation and oxidative stress associated with retinal degenerative diseases can be conveniently monitored by analyzing aqueous humor (AH) that is responsible for the nutrition of avascular structures of the eye. It is produced by active secretion from the non-pigmented epithelium of the ciliary body and can freely diffuse into the posterior cavity to wet the retina [31,32]. Similarly to the retina, AH is separated by the blood-AH barrier and, under normal conditions, it exhibits antioxidant properties and suppresses both innate and adaptive immunity [33,34]. However, oxidative and inflammatory processes that are associated with retinal degenerative diseases can manifest as an elevated concentration of the respective markers in AH. Indeed, alterations in malondialdehyde concentration and total antioxidant activity were found in AH of patients with glaucoma and exfoliation syndrome, as well as in animals with intraocular inflammation [35,36,37,38]. Furthermore, the increased levels of pro-inflammatory cytokines characterize AH of patients with AMD, diabetic retinopathy, macular edema, and glaucoma [39,40,41,42]. Meanwhile, alterations in oxylipin content of AH that are associated with LIRD and other retinal degenerative diseases remain mostly uninvestigated.

Previously, we have developed a rabbit model of LIRD reproducing many aspects of AMD and other retinal degenerative diseases, such as apoptosis of photoreceptors and migration of RPE cells, as well as the breakdown of the blood-retinal barrier, leukocyte infiltration, edema, and other signs of intraocular inflammation [3,14,43]. These effects were accompanied by oxidative stress, which was efficiently prevented by premedication while using mitochondria-targeted antioxidant SkQ1 [3]. In the present study, we employed this model of LIRD to get insight into oxidative and inflammatory processes contributing to retinal degeneration diseases, such as AMD. To this end, we performed pathomorphological and electrophysiological characterization of degenerative processes in the light-exposed retina and described the histological signs of the accompanying inflammation. We next examined changes in the content of inflammatory and oxidative stress markers in AH of the illuminated animals at different stages of LIRD. Moreover, while using the quantitative metabolomic (Ultra Performance Liquid Chromatography—Tandem Mass Spectrometry, UPLC-MS/MS) technique, we identified, for the first time, alterations in the AH patterns of different lipid mediators, such as phospholipid derivatives, polyunsaturated fatty acids (PUFAs), and oxylipins, which are associated with development of LIRD. The obtained data provide a rationale for using a combination of antioxidant and non-steroidal anti-inflammatory (NSAID) drugs for the prevention and treatment of oxidative stress and inflammation that are associated with LIRD. Consistently, we demonstrated that premedication of the eyes with mitochondria-targeted antioxidant SkQ1 with their subsequent treatment while using topical NSAID Nepafenac results in suppression of lipidomic signs of oxidative stress and inflammation and the overall improvement of the state and functionality of the illuminated retina. Therefore, the latter two-component approach was suggested to be of high potential in complex treatment of AMD and other retinal degenerative diseases.

## 2. Results

### 2.1. Clinical, Electrophysiological and Morphological Characteristics of the Rabbit Model of LIRD

In the first step, we monitored the clinical state of the rabbit eyes in the course of LIRD focusing on possible signs of the associated inflammation. To this end, the animals of the model group (see Section 4.3.) were subjected to slit lamp and indirect funduscopic examinations performed prior to the light exposure, immediately after the illumination, as well as on the 1, 3, and 7 day of the subsequent period. We observed an optically clean vitreous body and a moderately vascular retina at all of the time points. The blood vessels extended medially and laterally to the optic disk, and the vessels of the choroid were wide and straight. No signs of vitreous body clouding, exudate, thinning of the vessels, swelling of the optic nerve, hemorrhage, or retinal detachment were detected both prior and after the illumination. Thus, the eyes of the animals that were exposed to bright light exhibited no macroscopic signs of retinal inflammation or edema.

**Figure 1:**
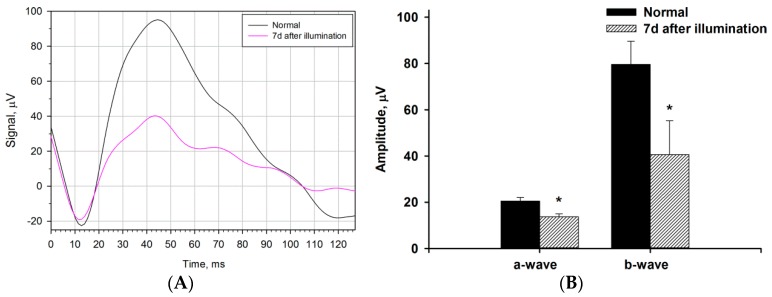
Alterations in electrophysiological activity of the rabbit retina under conditions of light-induced retinal degeneration (LIRD). The animals were illuminated with intense visible light (30,000 lx) for 3 h. (**A**) Representative scotopic electroretinograms recorded before (normal) and 7 days after the illumination. Recordings were made using white flashes at light intensity of 3 cd·s/m^2^. (**B**) Amplitudes of a- and b-waves of scotopic electroretinograms recorded before (normal) and seven days after the illumination. * *p* < 0.05 as compared with the respective parameters of the intact retina.

We next examined whether the bright light illumination induced general changes in the functional activity of the retina by electroretinographic (ERG) study conducted prior to the illumination and on the seventh day after the exposure. The retinograms were recorded under scotopic conditions by adapting animals to the dark and subsequent registration of ERG response to a flash (Figure 1). It was found that irradiation leads to a noticeable decrease in electrophysiological activity of the retina with similar alterations in both a- and b-waves (a decrease in amplitude and a slight increase in latency), reflecting the decrease in total responses of both photoreceptors and non-photoreceptor (predominantly bipolar) neurons of the retina.

**Figure 2:**
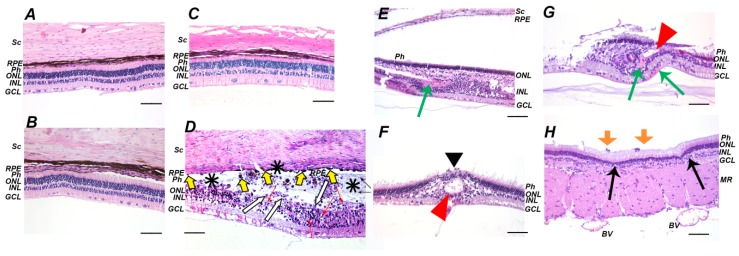
Histopathological findings in rabbit retina after the illumination with intense visible light (30,000 lx, 3 h). (**A**–**C**): before (**A**), immediately after (**B**) and one day after (**C**) the illumination retina demonstrates intact morphology. (**D**): acute signs of the retinal damage three days after the illumination. The signs include the presence of pycnotic nuclei and apoptotic bodies of photoreceptors (red arrows), retinal detachment from retinal pigment epithelium (RPE) (asterisks), activation of RPE cells (yellow arrows) and phagocytosis of apoptotic bodies by RPE cells (white arrows). (**E**–**H**): delayed signs of the retinal damage seven days after illumination. The signs include thinning of outer nuclear layer (ONL) and inner nuclear layer (INL) (**E**) and gliosis (**E**, green arrow), total loss of photoreceptors (**F**, black arrowhead), glial scar formation, and newly formed canals filled with edematous fluid (**F**, red arrowhead), Muller glia activation and hypertrophy and eosinophilic areas (**G**, green arrows), areas of ONL thinning (**H**, black arrows) and inflammatory cells between photoreceptor layer and RPE (**H**, orange arrows). Representative cross-sections of ocular fundus staining with hematoxylin and eosin; magnification 200×. Scale bar 20 μm. Retinal detachment at B and E is of artificial origin. Abbreviations: BV, blood vessel; GCL, ganglion cell layer; INL, inner nuclear retinal layer; MR, medullary ray; ONL, outer nuclear retinal layer; Ph, photoreceptor layer; RPE, retinal pigment epithelium; Sc, sclera.

We performed histological analysis of the posterior segment of the affected eyes to assess pathomorphological alterations underlying the revealed functional abnormalities of the illuminated retina. No differences were found between the state of the retina obtained immediately after the light exposure or on the next day and the retina of the intact animals (Figure 2A–C). However, three days after the illumination the multiple signs of the retinal damage were observed (Figure 2D). Generally, they included swelling and destruction of the outer segments of photoreceptors (Ph) and the cell death of photoreceptors and inner nuclear layer (INL) neurons (manifested as the formation of apoptotic bodies and their phagocytosis by macrophage-like cells), which results in a decrease in the thickness of these layers and the total thickness of the retina (up to subtotal or total retinal atrophy in some locations). There were also areas of retinal detachment with the formation of a space containing apoptotic bodies and photoreceptors fragments, some of which were phagocytized by activated RPE cells that migrated to these areas. Seven days after illumination, the acute phases of cell deaths were completed and the retina exhibited compensatory and regenerative processes (Figure 2E–H) manifested as an increase in retinal eosinophilia due to the activation and hypertrophy of Mueller glia. Importantly, the sites of damage (three day) and regeneration (seven day) were located focally over the retina, which is associated with its heterogeneous photosensitivity. Such character of the destruction might explain the absence of its macroscopic signs in the fundoscopic examination. Nevertheless, a general consequence of the irradiation can be defined as a decrease in the amount of cells over the entire retina, especially in the outer nuclear layer (ONL). Interestingly, the development of LIRD was associated with a histologically manifested inflammatory process. In particular, the areas of retinal atrophy and the RPE layer were in some places that were infiltrated by granulocytes and the choroid was characterized by increased cellularity and possessed enhanced fibroblast reproduction. In addition, the retina occasionally contained cysts and canals that were filled with edematous fluid. The inflammation seems to have a chronic character, as its signs maintained, even on the seventh day after the illumination.

We concluded that our model of LIRD involves not only loss of photoreceptors and other neurons and, consequently, the suppression of the retinal activity, but also the development of local inflammation and, thereby, can be employed for investigation of inflammatory mechanisms that are associated with retinal degenerative diseases.

### 2.2. Biochemical Manifestations of LIRD-Associated Inflammation in the Aqueous Humor

**Figure 3:**
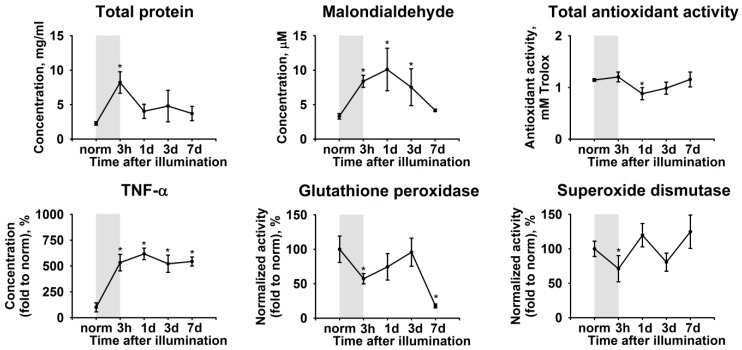
Alterations in biochemical properties of rabbit aqueous humor (AH) under conditions of LIRD. The animals were illuminated with intense visible light (30,000 lx) for 3 h (the course of the illumination is shown as gray box). AH samples were collected before (norm) and immediately after (3 h) the illumination as well as on 1, 3, and 7 day post-exposure. The samples were analyzed for total protein, tumor necrosis factor alpha (TNF-α), and malondialdehyde content as well as total antioxidant activity (in Trolox equivalent) and activity of glutathione peroxidase and superoxide dismutase using the respective assays. * *p* < 0.05 as compared to parameters of AH of the intact (control) animals.

Intraocular inflammation is known to manifest in AH by increased concentration of proteins, oxidative stress markers, and inflammatory molecules (see Introduction). As such, we assessed the biochemical alterations in AH, which are associated with the development of LIRD in experimental animals. AH collection and analysis were performed prior to the light exposure and during the next seven days. All of the samples exhibited an acute elevation of the total protein concentration (Figure 3). The protein concentration reached more than four-fold excess during the illumination and returned to the baseline control value within the next 24 h. This phenomenon was accompanied by elevation of concentration of TNF-α, the pro-inflammatory cytokine most commonly found in AH (Figure 3). Yet, in contrast to the total protein, the concentration of TNF-α was maintained at a high level until the seventh day, which indicated the development of chronic inflammation in accordance with our histological observations (see Figure 2).

Interestingly, all of these effects were accompanied by alterations in the redox state of AH. Thus, we detected an increase in the AH concentration of malondialdehyde, representing a product of lipid peroxidation. The highest level of this compound was detected on the first day after illumination, whereas, on the seventh day, its concentration normalized (Figure 3). The opposite dynamics was expectedly peculiar to the total antioxidant activity of AH (Figure 3). Finally, we observed a moderate decrease in AH concentration of common antioxidant enzymes, superoxide dismutase, and glutathione peroxidase, during the illumination. Within the next days, both of the enzymes demonstrated uptrend, although glutathione peroxidase exhibited a statistically significant decrease on the seventh day (Figure 3).

These data, taken together, indicate that LIRD is preceded and accompanied by oxidative stress and inflammatory reactions, the signs of which manifest in AH.

### 2.3. Lipidomic Signs of LIRD-Associated Inflammation in the Aqueous Humor

**Figure 4:**
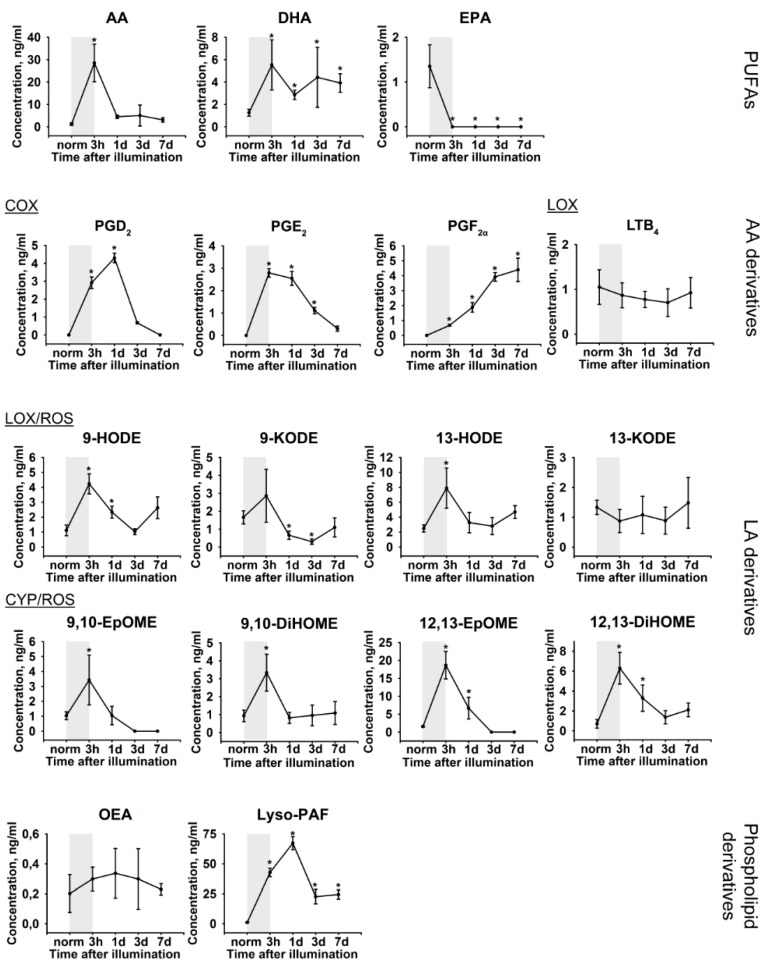
Alterations in patterns of lipid mediators in rabbit AH under conditions of LIRD. The animals were illuminated with intense visible light (30,000 lx) for 3 h (the course of the illumination is shown as gray box). AH samples were collected before (norm) and immediately after (3 h) the illumination as well as on 1, 3, and 7 day post-exposure. The concentrations of the lipid mediators (phospholipid derivatives, PUFAs and oxylipins) in aqueous AH were measured using quantitative UPLC-MS/MS analysis. * *p* > 0.05 as compared to the parameters of AH of the intact (control) animals. The identified oxylipins are divided into subgroups according to their origin and biosynthetic pathways, involving cyclooxygenases (COX), lipoxygenases (LOX), cytochrome P450 monooxygenases (CYP), or reactive oxygen species (ROS) (non-enzymatic pathway).

The mechanisms of the revealed LIRD-associated oxidative stress and inflammation can be determined by analyzing the patterns of lipid mediators, such as PUFAs and their oxidized derivatives, oxylipins. Therefore, we performed quantitative mass-spectrometry studies of the lipids extracted from AH samples, which were obtained immediately before and immediately after illumination of the animals, as well as on the 1, 3 and 7 day post-exposure. UPLC-MS/MS analysis identified a total of 17 lipid compounds (Figure 4), including three PUFAs (AA, docosahexaenoic acids (DHA), and eicosapentaenoic acid (EPA)), two phospholipid derivatives ((Z)-N-(2-Hydroxyethyl) octadec-9-enamide (oleoylethanolamine, OEA), 1-O-hexadecyl-sn-glyceryl-3-phosphorylcholine (lyso-Platelet-Activating Factor, lyso-PAF)), and twelve oxylipins. The latter compounds included four AA derivatives (prostaglandin E2 (PGE_2_), prostaglandin D2 (PGD_2_), prostaglandin F_2a_ (PGF_2α_), and leukotriene B4 (LTB_4_)), and eight linoleic acid (LA) derivatives (9-/13-hydroxyoctadecadienoic acids (9-HODE/13-HODE), 9-/13-oxo-octadecadienoic acids (9-KODE/13-KODE), 9,10-/12,13-epoxyoctadecamonoenoic acids (9,10-EpOME/12,13-EpOME), and 9,10-/12,13- dihydroxyoctadecamonoenoic acids (9,10-DiHOME/12,13-DiHOME)). In our LIRD model, we revealed a short-term increase in the AH content of AA and DH and a decline in levels of EPA during the illumination (indicated in Figure 4 as gray boxes). In addition, we detected a more prolonged increase in the concentration of Lyso-PAF. These short-term responses (except for EPA) mostly resolved within the next 1–3 days. Remarkably, there were obvious trends in the dynamics of oxylipins content during the development of LIRD. The most pronounced changes were found in prostaglandins PGE_2_, PGD_2_, and PGF_2α_ demonstrating a long-term increase, which, in the case of PGF_2α_, did not resolve, even at the seventh day post-illumination. By contrast, almost all of the LA derivatives exhibited a short burst during the light exposure and their concentration tended to recover in one day. Notably, whereas prostaglandins are biosynthesized mostly via COX-dependent pathway, the detected LA derivatives can be generated not only by enzymatic (LOX-dependent and CYP-dependent), but also by non-enzymatic mechanisms that involve ROS (the respective indications are given in Figure 4). Thus, one can expect that LIRD-associated inflammation can be responsive to a treatment while using COX inhibitors (such as NSAIDs) and antioxidants.

### 2.4. Functional and Morphological State of the Retina in the Course of Combined Antioxidant/Anti-Inflammatory Treatment of LIRD-Associated Inflammation

We next analyzed the functional and morphological state of the retina in four groups of experimental animals with the induced model of LIRD to confirm our conclusions regarding mechanisms of LIRD-associated inflammation and to find new routes for its prevention and treatment (See Section 4.3.). The animals of the first group were subjected to the illumination without any subsequent treatment. In the second group, the animals were premedicated prior to the illumination with mitochondria-targeted antioxidant SkQ1, according to the method that was developed in our previous study [3]. The animals of the third group were treated for seven days after the illumination with Nepafenac, representing topical NSAID that is commonly used for the treatment of ocular inflammation [44]. Finally, the rabbits of the fourth group received combined therapy involving the administration of both the antioxidant and the NSAID.

**Figure 5:**
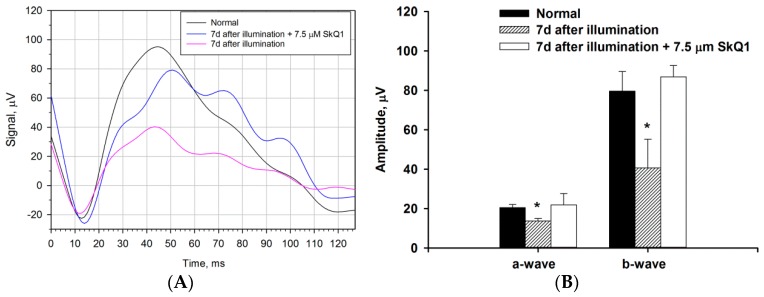
Electrophysiological activity of the rabbit retina under conditions of LIRD with (blue) or without (pink) premedication using SkQ1. The animals were illuminated with intense visible light (30,000 lx) for 3 h. (**A**) Representative scotopic electroretinograms recorded before (black) and 7 days after the illumination. Recordings were made using white flashes at light intensity of 3 cd·s/m^2^. (**B**) Amplitudes of a- and b-waves of scotopic electroretinograms recorded before (normal) and 7 days after the illumination. * *p* < 0.05 as compared with the respective parameters of the intact retina.

ERG studies revealed that premedication with SkQ1 efficiently preserved normal electrophysiological activity of the retina in light-exposed animals (Figure 5A). Indeed, the amplitudes of both a-wave and b-wave of the retinogram corresponded to that of the healthy retina (Figure 5B). The treatment of animals with Nepafenac on the background of premedication with SkQ1 did not give a noticeable additive effect in ERG (data not shown), apparently due to almost the complete protection of the retinal neurons by SkQ1.

**Figure 6:**
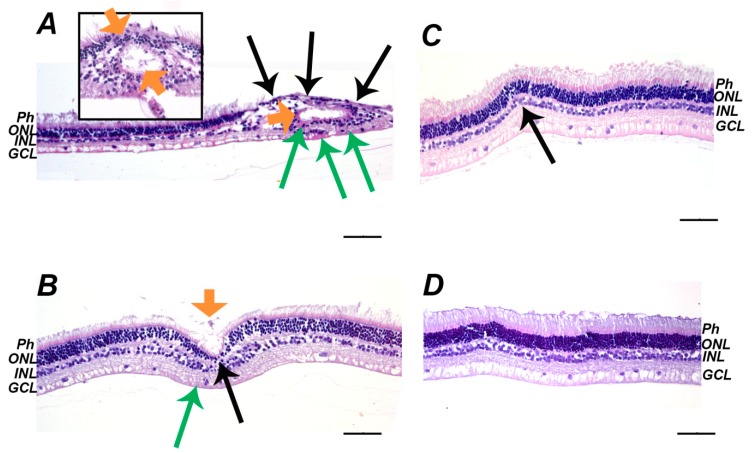
Histopathological findings in rabbit retina seven days after the illumination with intense visible light (30,000 lx, 3 h) without treatment (**A**), after premedication with SkQ1 (**B**), after treatment with Nepafenac (**C**), and after premedication/treatment using the combination of the both drugs (**D**). (**A**): The retina demonstrates severe signs of damage including thinning of ONL and INL and total loss of photoreceptors (black arrows), gliosis, glial scar formation and formation of canals filled with edematous fluid (orange arrows) as well as Muller glia activation and hypertrophy (eosinophilic areas, green arrows). (**B**): The retina exhibits much less prominent damage (thinning of ONL and INL–black arrow, glial activation–green arrow), but signs of pre-existent inflammation are present (debris in semi-canal, orange arrow). (**C**): The retina shows thinning of ONL (black arrow) without signs of inflammation. (**D**): No signs of the retinal damage and/or inflammation. Representative cross-sections of retina, staining with hematoxylin and eosin; magnification 200× (400× on inset). Scale bar 20 μm. Abbreviations: GCL, ganglion cell layer; INL, inner nuclear retinal layer; ONL, outer nuclear retinal layer; Ph, photoreceptor layer.

According to histological study, on the seventh day after irradiation, the retina of the animals that were premedicated with SkQ1 contains much less prominent areas of atrophy, activation of Müller glia and remodeling processes (Figure 6B) than non-premedicated retina (Figure 6A). However, the signs of pre-existed inflammation were still occasionally observed in these animals (Figure 6B, orange arrow). By contrast, no such signs were found in rabbits, which were additionally treated with Nepafenac (Figure 6D). Interestingly, the retina of the animals that solely received Nepafenac treatment was characterized by loci of the decreased thickness of the ONL, although inflammatory infiltration and edema were completely absent in this case (Figure 6C). Taken together, these data demonstrate that both SkQ1 and Nepafenac produce therapeutic effects in LIRD, and these effects are synergistic.

### 2.5. Biochemical and Lipidomic Alterations in the Aqueous Humor in the Course of Combined Antioxidant/Anti-Inflammatory Treatment of LIRD-Associated Inflammation

**Figure 7:**
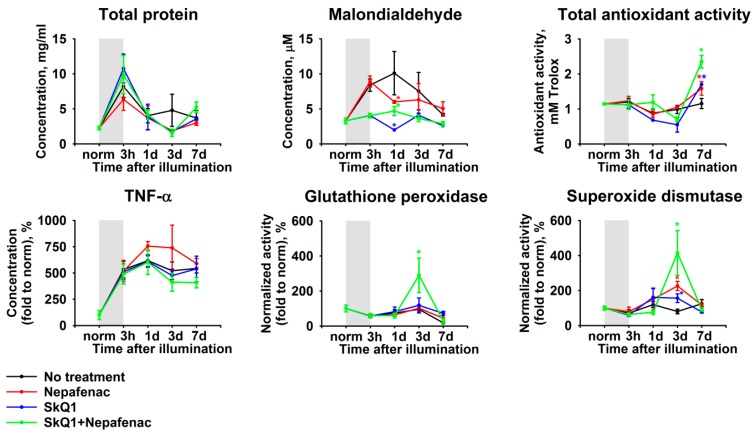
Alterations in biochemical properties of rabbit AH under conditions of LIRD without treatment (black), in the course of treatment with Nepafenac (red), after premedication with SkQ1 (blue), and in the course of the therapy using the combination of the both drugs (green). The animals were illuminated with intense visible light (30,000 lx) for 3 h (the course of the illumination is shown as a gray box). The AH samples were collected before (norm) and immediately after (3 h) the illumination as well as on 1, 3, and 7 day post-exposure. The samples were analyzed for TNF-α and malondialdehyde content as well as total antioxidant activity (in Trolox equivalent) and activity of glutathione peroxidase and superoxide dismutase using the respective assays. * *p* > 0.05 as compared to the parameters of AH of the untreated illuminated animals.

Biochemical analysis of AH samples that were collected from the rabbits of same groups (see previous section) revealed that all of the variants of the therapy did not have any reliable acute (short-term) effects, such as increase in total protein concentration, but demonstrated a delayed benefit in respect to recovery of almost all of its parameters (Figure 7). Particularly, in the presence of premedication/treatment, we observed the suppression of malondialdehyde (peak on one day) and the increase in total antioxidant activity (peak on seven day), as well as pronounced elevation of superoxide dismutase and glutathione peroxidase activities (peak on three day). An exception was TNF-α, which exhibited a slight increase during Nepafenac treatment, although it was statistically insignificant. Notably, in all cases, the most prominent effects were observed in the case of the combined treatment, which indicated synergistic mechanisms of action of SkQ1 and Nepafenac towards LIRD-associated inflammation.

**Figure 8:**
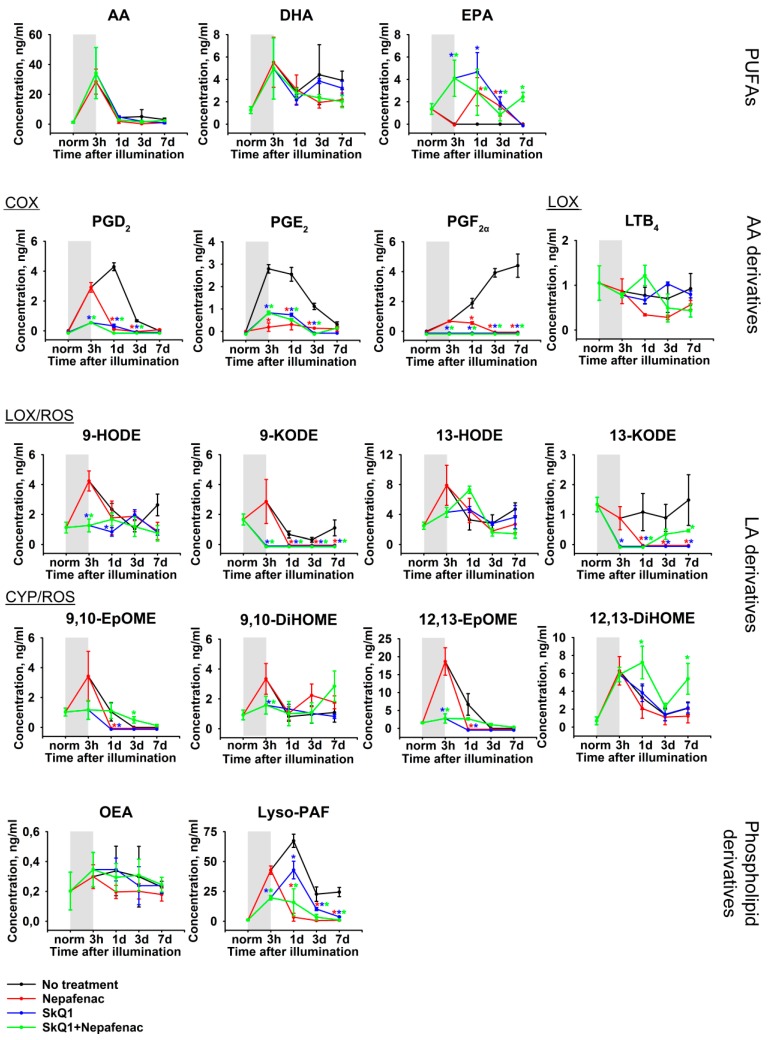
Alterations in patterns of lipid mediators in rabbit AH under conditions of LIRD without treatment (black), in the course of treatment with Nepafenac (red), after premedication with SkQ1 (blue), and in the course of the therapy using the combination of the both drugs (green). The animals were illuminated with intense visible light (30,000 lx) for 3 h (the course of the illumination is shown as gray box). The AH samples were colected before (norm) and immediately after (3 h) the illumination as well as on 1, 3, and 7 day post-exposure. The concentrations of the lipid mediators (phospholipid derivatives, PUFAs and oxylipins) in AH were measured using quantitative UPLC-MS/MS analysis. * *p* > 0.05 as compared to the parameters of AH of the untreated illuminated animals. The identified oxylipins are divided into subgroups according to their origin and biosynthetic pathways, involving COX, LOX, CYP, or ROS (non-enzymatic pathway).

However, most of the information regarding these mechanisms was obtained through analysis of lipidomic alterations in AH of the animals from the abovementioned groups (Figure 8). Nepafenac expectedly downregulated all of the COX-generated oxylipins (prostaglandins), starting from the very begin of the treatment (the first day after illumination). In the same time, premedication with SkQ1 produced comparable suppressive effects on these oxylipins, although the combined therapy was somewhat more effective. By contrast, AH concentration of LA-derived oxylipins was only affected by the antioxidant premedication and the suppressive effect of the combined therapy was seemingly solely provided by SkQ1. Interestingly, neither of the secreted PUFAs were affected by any kind of treatment. At the same time, Nepafenac potently downregulated lyso-PAF and this effect seems to be specific to this NSAID, as it cannot be substituted by SkQ1.

Taken together, our data confirm that LIRD is preceded and accompanied by oxidative stress, as well as inflammation mainly governed by COX-dependent pathways and consistently sensitive to NSAID Nepafenac. Both mechanisms can be suppressed by SkQ1 exhibiting not only antioxidant, but also anti-inflammatory effects and, therefore, strongly protecting the structure and electrophysiological activity of the retina. Meanwhile, Nepafenac possesses specific anti-inflammatory effects and, therefore, complements SkQ1 protection, as evidenced by histological data. These findings provide a rationale for the inclusion of a combination of both drugs in the therapy of LIRD-associated inflammation.

## 3. Discussion

Growing evidence indicates that inflammation contributes to the pathogenesis of a number of blind-causing retinal degenerative diseases, such as AMD or photic maculopathy [45]. The aim of the present study was to identify inflammatory mechanisms that are associated with retinal degeneration and, based on these data, suggest possible routes for its prevention and treatment. We used a rabbit model of LIRD that was previously developed in our laboratory to reach this aim [3,14,15]. Indeed, there are a number of important similarities in ocular morphology and biochemistry between human and rabbit eyes, which make rabbits attractive species for modeling ocular diseases [9]. The animal models of LIRD are generally acknowledged for AMD studies as they share many common features with the human disease (discussed in [3]). Consistently, we previously demonstrated that exposure of the eyes of experimental animals (rats and rabbits) to different doses of bright visible light leads to the development of retinal degenerative processes, including the apoptosis of photoreceptors and other retinal neurons, as well as vacuolization of RPE cells, their phagocytic activity, and migration into the neural retina, representing common hallmarks of AMD [3,14,15,43]. In the present study, we used a rabbit model of LIRD that was induced by intense short-term illumination (30,000 lx for 3 h) [3,15]. We complemented its histological characterization with electrophysiological (ERG) data in order to more accurately describe the pathological process. It was found that light irradiation of the rabbit eye indeed suppresses the functionality of the retina by affecting both a- and b-waves of ERG. These data reflect a decrease in total responses of both photoreceptors and non-photoreceptor (predominantly bipolar) neurons of the retina, which is in good agreement with our pathomorphological findings.

Interestingly, in previous LIRD studies, we have observed that focal signs of inflammation including disturbance in the blood-retinal barrier and inflammatory infiltration accompany the development of the degenerative changes in the retina. Indeed, retinal edema was found to already emerge at the early stages of LIRD, gradually expanding in the course of the disease and becoming complemented by granulocytic infiltration [14,43]. All of these signs were occasionally found in the model used in the current work, indicating that intraocular inflammation is a sustainable complication of LIRD. Importantly, similar inflammatory processes were observed in AMD [45]. Thus, along with biochemical signs, such as the presence of inflammatory components in drusen, both early and atrophic forms of AMD can exhibit acute inflammation symptoms, including increased infiltration of inflammatory cells in the retina [18,46]. In turn, wet AMD is associated with the breakdown of the blood-retinal barrier, which promotes neovascularization and blood leakage from the abnormal vessels causing edema and detachment of the pigment epithelium and the retina [19,45,47,48]. In our LIRD model, we observed all of these signs, although they emerged focally, were of low severity and, therefore, did not manifest in the funduscopic examination. It should be added that, despite the absence of drusen in our model, it is characterized by extensive oxidative stress in the retina and RPE [3], which is known to play a crucial role in drusen formation and AMD (see Introduction section). Overall, the rabbit model of LIRD that is employed in this study reproduces different routs of AMD pathogenesis, including those that are related to oxidative stress and inflammation and, may, therefore, be used for trialing of the appropriate therapies.

As it was stated above, analyzing the content of the appropriate markers in AH can be used to monitor the inflammatory reactions in the retina and the neighboring tissues. Although AH sampling is an invasive procedure, it is still accomplishable in humans in contrast to any of the tissue analyses, thereby supporting the diagnostic value of AH. The increased levels of pro-inflammatory cytokines in AH were previously found, not only in patients with pronounced intraocular inflammation (such as posterior uveitis), but also in individuals with AMD, diabetic retinopathy, macular edema, or glaucoma [39,40,41,42]. In the current study, we have demonstrated that the development of LIRD is accompanied by a chronic increase in the AH content of pro-inflammatory cytokine TNF-α. The latter was shown to accumulate in light-exposed rabbit retina [23] and, may, therefore, be secreted to AH. Furthermore, the levels of this cytokine were demonstrated to be elevated in AH of patients with dry or treated wet forms of AMD and diabetic retinopathy, but not glaucoma [41,42,49]. Thus, this cytokine can be considered as a specific AH marker of the inflammation, being associated with LIRD and similar retinal degenerative conditions. It should be added that anti TNF-α drugs were recently approved for the treatment of ocular inflammation [50].

Total protein concentration in AH is another well-known parameter characterizing intraocular inflammation. Inflammatory processes lead to the disruption of the blood-ocular barrier and enter of plasma proteins in the eye tissues, including AH. In the models of pronounced intraocular inflammation, such as endotoxin-induced uveitis, the total protein concentration in AH rapidly increases 15–30-fold and only resolves in seven days [36,37]. In our study, we observed only ~4-fold increase of this parameter and its recovery within 24 h, which confirms a moderate character of LIRD-associated inflammation.

The crucial factor of LIRD is oxidative stress that is induced by multiple photochemical reactions occurring in the illuminated retina [16]. The rabbit model of LIRD, which was used in this study, was previously characterized in terms of the redox status of the retina. Oxidative stress was shown to manifest as a short-term increase in the retinal content of malondialdehyde and hydrogen peroxide [3]. In the current work, we expectedly monitored an increase in malondialdehyde in AH, which might reflect its accumulation in the illuminated degenerating retina. Consistently, the enhanced level of malondialdehyde in AH was demonstrated to accompany ocular inflammation [37]. Somewhat different trends were observed regarding total antioxidant activity, which moderately decreased in AH, in contrast to dramatic elevation in the illuminated retina [3]. Yet, this discrepancy might be explained by the fact that absolute antioxidant activity of AH is very high (it is highly enriched in ascorbate and other antioxidant molecules [51]) and, therefore, it is less affected by oxidative stress in neighboring tissues. Finally, we detected a decrease in AH activity of common antioxidant enzymes, superoxide dismutase, and glutathione peroxidase, which is characteristic for LIRD and endotoxin-induced ocular inflammation [23,37]. In the aggregate, all of these data confirm that oxidative stress is a hallmark of LIRD and the accompanying inflammatory responses occur on the background of oxidative processes, the signs of which can be detected in AH.

Along with pro-inflammatory cytokine and biochemical markers of oxidative stress, while using a high-resolution quantitative lipidomic technique, we managed to reveal alterations in the AH patterns of lipid mediators, such as phospholipid derivatives, PUFAs, and oxylipins. The first principle effect that was identified in this study was a short-term increase in the concentration of AA and DHA, long-term decrease in levels of EPA, and long-term elevation of concentration of lyso-PAF and prostaglandins. These alterations are coherent with the upregulation of TNF-α and seem to reflect the mechanism of LIRD-associated inflammation. EPA generally possesses anti-inflammatory activity; it is present in AH at relatively low concentrations [52] and, therefore, expectedly decreases in response to LIRD-associated inflammation. DHA and its metabolites normally also exhibit anti-inflammatory activity, but this PUFA is present the retina in extremely high amounts and it plays specific roles in the structure and function of this tissue. In particular, DHA is released during oxidative stress to be converted into neuroprotective compounds, such as neuroprotectin D1 [53]. Thus, the increase in the AH concentration of DHA seems to represent the response of the retina to light-induced oxidative stress and degeneration, rather than inflammatory reaction. By contrast, AA has well-known pro-inflammatory properties and it has been implicated in triggering intraocular inflammation. Thus, it becomes released in response to the action of platelet-activating factor (PAF), which induces the generation of prostaglandins (both by upregulating AA and activating COX enzymes [54]), thereby promoting vascular permeability and macular edema [55]. Interestingly, intraocular inflammation can be induced not only by PAF, but also by its precursor/metabolite lyso-PAF taken in a similar dose [56]. Consistently, in our model, LIRD is associated with prolonged upregulation of lyso-PAF. Furthermore, PAF can be produced from lyso-PAF (by Lyso-PAF:acetyl-coenzyme A acetyltransferase) or from lysophosphatidyl choline (lyso-PC; biosynthesized by phospolipase A2), the concentration of which also increases in AH during ocular inflammation [57]. Given all of these data, it is not surprising that, in our model, LIRD is associated with prolonged growth in AH concentration of prostaglandins, among which PGF_2α_ remained elevated, even at the seventh day post-exposure. Indeed, LIRD is known to be accompanied by an increase in expression levels of COX-2 in the retina [23,24]. A similar growth of AH concentration of prostaglandins was observed in rabbit models of endotoxin-induced uveitis, BSA-induced uveitis, paracentesis, laser irradiation, etc. [58,59,60,61], which indicated that the above-described inflammatory mechanism is common for intraocular inflammation of different origins.

According to our data, the signs of LIRD-associated inflammation can be effectively suppressed by the treatment of the eyes with ocular NSAID Nepafenac. This drug represents a precursor of Amfenac, which inhibits the activity of COX enzymes and thereby downregulates the synthesis of prostaglandins in the eye. Nepafenac is metabolized into amfenac in the ciliary body, choroid, and retina, and it is suggested for the treatment of inflammatory component of AMD and other retinal degenerative diseases [44,62]. The administration of Nepafenac in the rabbit eye was shown to reduce prostaglandin concentration in AH [63], which is in good agreement with our findings. Remarkably, in our LIRD model, Nepafenac also strongly reduced the AH level of lyso-PAF and significantly upregulated EPA. We can conclude that the effect of Nepafenac can be derived from regulating these mediators in addition to inhibiting the synthesis of prostaglandins, given the crucial role of lyso-PAF in promoting intraocular inflammation (see above), as well as anti-inflammatory activity of EPA. These novel findings add more insights to the general mechanism of the pharmaceutical activity of Nepafenac.

The second principle effect that is identified in our lipidomic study is LIRD-associated short burst in AH content of linoleic acid derivatives HODE/KODE and EpOME/DiHOME, which resolves as fast as during 24 h after the illumination. These oxylipins possess both pro-inflammatory and anti-inflammatory activities and can be synthesized from LA via LOX- and CYP-dependent pathways, respectively [26]. Yet, in contrast to prostaglandins, they can also be produced by non-enzymatic mechanisms via the oxidation of PUFAs by free radicals [64]. While considering the fast dynamic changes in oxidative stress markers in the light-illuminated retina [3] and AH (this study), we suppose that the detected increase in LA derivatives mainly reflects LIRD-associated oxidative stress. Indeed, the retina is characterized by the highest content of PUFAs in addition to the highest metabolic rate and oxygen consumption among the human tissues and, therefore, it is extremely vulnerable to lipid peroxidation yielding corresponding oxylipins [16,28]. Furthermore, the accumulation of total LA-derivative HODEs in the retina was previously shown to accompany LIRD [29], which can underlie their secretion in AH. Notably, all of the effects of LIRD on AH concentration of LA-derived oxylipins are similarly inhibited by premedication of the rabbit eyes with mitochondria-targeted antioxidant SkQ1, whereas Nepafenac treatment was expectedly unfunctional in this respect. Indeed, SkQ1 was recently demonstrated to efficiently suppress LIRD by preventing light-induced oxidative stress, including lipid peroxidation [3]. It should be added that the inhibition of EpOME/DiHOME synthesis/release by SkQ1 is an important additional activity of the latter with respect to general protection of mitochondria, since these oxylipins are known to cause mitochondrial dysfunction [26].

Interestingly, along with suppressing LA-derived oxylipins, premedication with SkQ1 potently inhibits the growth of prostaglandins and produces an ant-inflammatory effect by upregulating EPA. There are data indicating that inflammation, being associated with retinal degeneration diseases, might be promoted by oxidative stress [65]. Thus, LIRD and associated inflammatory reactions can be suppressed by different antioxidants, such as resveratrol, celastrol, edaravone, etc. [65,66,67]. Yet, the mechanisms that underlie these relationships are yet to be fully understood. It should be emphasized that, in contrast to Nepafenac, SkQ1 does not restore normal AH level of lyso-PAF, which plays a central role in triggering LIRD-associated inflammation. In addition, it displays no effect on the antioxidant enzymes in AH. In our model of LIRD, the most prominent antioxidant and anti-inflammatory benefits were achieved by simultaneously administrating both SkQ1 and Nepafenac. Thus, the prominent neuroprotective activity of SkQ1 was supported by specific anti-inflammatory effects of Nepafenac. These findings the provide rationale for using such combined therapy with respect to LIRD, as well as AMD and other retinal degenerative diseases.

## 4. Materials and Methods

### 4.1. Materials

The Institute of Mitoengineering of Moscow State University (Moscow, Russia) provided SkQ1 (10-(60-plastoquinonyl)-decyltriphenylphosphonium). Nepafenac (Nevanac), Alcaine and Betadine Ophthalmic were from Alcone (New York, NY, USA). Tiletamine and zolazepam were from Virbac (Carros, France). Xylazine hydrochloride was from Nita-Farm (Saratov Oblast, Russia). Phosphate buffer saline (PBS) was from Thermo Fisher Scientific (Waltham, MA, USA). Trolox (6-hydroxy-2, 5, 7, 8-tetramethylchroman-2-carboxylic acid) was from Sigma–Aldrich (St. Louis, MO, USA). TNF-α assay kit was from Cloud-Clone Corp. (Katy, TX, USA). Reagents for histological examination were from Biovitrum (Moscow, Russia). Malondialdehyde and bicinchoninic acid assay kits were from Sigma-Aldrich (USA). Glutathione peroxidase and superoxide dismutase assay kits were from Randox (Crumlin, UK). Oasis® PRIME HLB cartridge (60 mg, 3cc, cat.no. 186008056) were obtained from Waters (Eschborn, Germany). Other reagents and supplies were from Sigma–Aldrich, Amresco (St. Louis, MO, USA), and Serva (Heidelberg, Germany). All of the buffers were prepared while using ultrapure deionized water.

### 4.2. Animals and Ethics Statement

The study involved 83 pigmented male rabbits six months old (2.3 to 3 kg) that were purchased from a certified farm (Krolinfo, Moscow, Russia). The animals were housed in individual cages at a 12 h light-dark cycle at a temperature of 22–25 °C and humidity of 55–60% with free access to maintenance rabbit food and water. The health status of the animals was monitored daily and no adverse events were observed. All of the experiments were performed under general anesthesia induced with intramuscular injection of 1:2 mixture of 50 mg/mL tiletamine/zolazepam and 20 mg/mL xylazine hydrochloride. Prior to AH collection, the animals were additionally instilled with topical anesthetic Alcaine. For the histological analysis of the posterior segment of the eye, the animals were humanely euthanized by an overdose of the anesthetic. The enucleating of the eyeballs was performed postmortem. After the other experiments, the animals were rehabilitated for three days and returned to the farm. The treatment of the animals was performed according to the 8th edition “Guide for the Care and Use of Laboratory Animals” of the National Research Council and “Statement for the Use of Animals in Ophthalmic and Visual Research” of The Association for Research in Vision and Ophthalmology (ARVO). The Belozersky Institute of Physico-chemical Biology Animal Care and Use Committee approved the protocol (Protocol number 1/2016).

### 4.3. Experimental Model

The experiments were performed while using a single-blind method. Before the experiments, the rabbits were housed as described above for one week for acclimatization. The animals were divided into six groups, including absolute control (seven animals), model (12 animals), and four experimental groups (groups 1–4, 16 animals in each group). The absolute control group contained intact animals, kept during the experiment under the normal conditions described above. The animals of the model and experimental groups were exposed to bright visible light (30,000 lx, 3 h, 0.15 W/cm^2^) under general anesthesia, exactly as described in our previous study [3]. Eight hours before the illumination the all animals (including the absolute control group) were kept on a diet with only free access to water. Immediately after the illumination, or after 1, 3, or 7 days post exposure (during these time periods the animals were housed under normal conditions described above), the animals of the model group were subjected to clinical and ERG examination (ERG was performed prior to illumination and on the seventh day), and then sacrificed for histological analysis. The animals of the experimental groups were left without treatment (group 1), or premedicated with SkQ1 prior to illumination (group 2), or treated with Nepafenac for seven days after the illumination (group 3) or premedicated with SkQ1 and treated with Nepafenac for seven days after the illumination (group 4). Premedication with SkQ1 included six subsequent conjunctival instillations (one instillation per 10 min.) of 50 μL of 7.5 μM SkQ1 in each eye, as described in [3]. Treatment with Nepafenac included conjunctival instillations of 50 μL of Nevanac in each eye three times a day. Immediately after the illumination or 1, 3, or 7 days post exposure the animals of the experimental groups were subjected to clinical examination and ERG, as well as a procedure of AH collection, as described below. Three animals from each experimental group were euthanized and posterior segments of their eyes were subjected to histological analysis.

### 4.4. Clinical and Electrophysiological Examination

Clinical examination was performed while using a slit lamp (Heine, Herrsching am Ammersee, Germany) and a fundus camera (Kowa Genesis-D, Tokyo, Japan). The possible changes in the cornea, lens, vitreous, retina, and optic nerve were evaluated.

The standard scotopic ERG was recorded using visual electrophysiology system RETIport ERG (An-vision, Germany). Prior to the examination, the animals were dark-adapted for 1 h. The recordings were performed under conditions of general anesthesia (see Section 4.2.), topical anesthesia (Alcaine), and mydriasis (1% tropicamide). The active gold wire Koijman electrode was placed (via a contact lens) to the central cornea, whereas the needle ground and negative electrodes were subcutaneously positioned in the tissue 0.5 cm from the lateral angle of the eye and in the forehead tissue, respectively. Scotopic recordings were made while using white LED flashes (7000 K) at light intensity of 3 cd·s/m^2^ with frequency of 0.3 Hz. The interferences were removed, the data were processed while using the software provided with the ERG system and visualized using SigmaPlot 11 (Systat Software, San Jose, CA, USA). The ERG analysis was made on amplitude of the a-wave and b-wave.

### 4.5. Histology

Freshly enucleated whole rabbit eyes were fixed in 4% buffered formaldehyde (pH 7.4) for histological analysis and pathomorphological assessment. After the fixation, the anterior segment and vitreous were removed, and the posterior eye chambers containing retina, retinal pigment epithelium, choroid, and sclera were isolated. The preparations of posterior eye chamber were subjected to routine histological processing, including immersing specimens in a series of ethyl alcohol solutions. Properly the oriented preparations were embedded in paraffin, and 4 µm-thick midsagittal sections were obtained from each paraffin block. The sections were mounted on slides, deparaffinized, rehydrated, stained with Carazzi’s hematoxylin and eosin Y, and examined while using LEICA DM 4000B (Leica, Wetzlar, Germany) microscope. The microphotographs were obtained while using Leica DFC400 digital camera (Leica, Wetzlar, Germany). Processing of the microphotographs was performed using the AxioVision 8.0 (Carl Zeiss, Oberkochen, Germany) and Adobe Photoshop CS6 Extended (Adobe Systems, San Jose, CA, USA) software.

### 4.6. Aqueous Humor Collection

AH was collected under general anesthesia (see Section 2.2.). Prior to the procedure, the eyes were instilled with topical anesthetic Alcaine and irrigated with antiseptic Betadine Ophthalmic. 100–150 µL of AH was collected while using 30G needle and insulin syringe and aliquoted in sterile plastic tubes. For UPLC-MS/MS analysis, 50 µL of AH were mixed with 1 µL of 0.05% butylated hydroxytoluene. All of the samples were stored at −70 °C until biochemical or lipidomic examination.

### 4.7. Total Protein Concentration Measurement

Protein concentration in the AH samples was measured while using bicinchoninic acid assay. Colorimetric reaction was monitored with Synergy H4 Hybrid plate reader (Biotek, Winooski, VT, USA). The calibration curve was constructed using 0.025–2 mg/mL bovine serum albumin solutions in PBS.

### 4.8. Lipid Peroxidation Analysis

Measuring malondialdehyde content in AH samples by standardized thiobarbituric acid assay, while using commercially available reagent kit, was utilized to assess the level of lipid peroxidation. Colorimetric reaction was monitored with Ultrospec 1000 spectrophotometer (Pharmacia, Uppsala, Sweden).

### 4.9. Total Antioxidant Activity Analysis

The total antioxidant activity in AH was analyzed via hemoglobin/H_2_O_2_/luminol system, as described in our previous works [68,69,70]. Briefly, the AH samples were diluted 200-fold with PBS and added to the reaction mixture, containing 0.01 mm luminol and 0.5 mm hemoglobin. The reaction was then induced by the addition of H_2_O_2_ to the final concentration of 6 μm. After brief vortexing, the samples were placed in Glomax-Multi Detection System luminometer (Promega, Madison, WI, USA) and chemiluminescence was monitored every one second for 10 min. The calibration was performed while using 1–8 μm standard solutions of Trolox and the results were presented in the Trolox equivalent.

### 4.10. Antioxidant Enzyme Activity Analysis

Superoxide dismutase and glutathione peroxidase activity in AH samples was measured by standardized tests while using commercially available kits. The acquired data were normalized to the total amount of protein in AH. All of the reactions were spectrophotometrically monitored using Synergy H4 Hybrid Reader (Biotek, Winooski, VT, USA).

### 4.11. TNF-α Concentration Measurement

The cytokine content in the samples of AH was assessed by ELISA, while using commercially available kit. Synergy H4 Hybrid Reader was utilized to measure the intensity of colorimetric reaction.

### 4.12. Lipid Extraction

The AH specimens were diluted with ice-cold anhydrous methanol, mixed with 2 ng of deuterated internal standard solutions, and centrifuged (12,000× *g*, 3 min.), mixed with 6 mL of 0.1% acetic acid and loaded onto solid-phase lipid extraction (Oasis^®^PRIME HLB cartridge (60 mg, 3cc)). The cartridge was washed with 2 mL of wash solution (15% methanol, 0.1% formic acid), and the lipids were sequentially eluted with 500 μL of anhydrous methanol and 500 μL of acetonitrile. The resulting samples were mixed, concentrated under gentle stream of nitrogen, and stored at −70 °C until UPLC-MS/MS analysis.

### 4.13. UPLC-MS/MS Analysis

For the identification of lipid mediators, the respective lipid extracts were analyzed while using 8040 series UPLC-MS/MS mass spectrometer (Shimadzu, Kyoto, Japan) in multiple-reaction monitoring mode at a unit mass resolution for both the precursor and product ions, as described previously [71]. Comprehensive analysis of lipid metabolites was performed by using a composition of internal standards (tetranor-PGEM-d6 (cat.no. 314840), 6-keto PGF1α-d4 (cat.no. 315210), TXB2-d4 (cat.no. 319030), PGF2α-d4 (cat.no. 316010), PGE2-d4 (cat.no. 314010), PGD2-d4 (cat.no. 312010), Leukotriene (LT) C4-d5 (cat.no. 10006198), LTB4-d4 (cat.no. 320110), 5(S)-HETE-d8 (cat.no. 334230), 12(S)-HETE-d8 (cat.no. 334570), 15(S)-HETE-d8 (cat.no. 334720), PAF C16-d4 (cat.no. 10010229), Oleoyl Ethanolamide-d4 (cat.no. 9000552), PGA2-d4 (cat.no. 310210) (Cayman Chemical, Ann Arbor, MI, USA), and a commercial software method package for lipid mediators (Lipid Mediator Version 2 software package Shimadzu, Kyoto, Japan), according to the manufacturer’s instructions.

### 4.14. Statistical Analysis

The data were analyzed by the mean standard deviation (SD) method, while using SigmaPlot 11 for calculation and visualization. The Mann–Whitney U test was applied for an estimation of statistical significance.

## 5. Conclusions

In this study, we ascertained the mechanisms of intraocular inflammation associated with LIRD in the rabbit model. Histologically, LIRD manifested as apoptosis of photoreceptors and other retinal neurons, as well as the vacuolization of RPE cells, their phagocytic activity, and migration into the neural retina. Pathomorphological changes were accompanied by the suppressed functionality of the retina, as evidenced by a decrease in ERG responses of both photoreceptors and non-photoreceptor neurons of the retina. All of these alterations were preceded and accompanied by oxidative stress and inflammation, as evidenced by granulocyte infiltration and edema in this tissue as well as the upregulation of total protein, pro-inflammatory cytokine, and oxidative stress markers (increased lipid peroxidation and decreased antioxidant activity) in AH. Quantitative lipidomic studies of AH elucidated neuroprotective (increased DHA) and inflammatory responses, including an increase in pro-inflammatory mediators AA and lyso-PAF, decrease in anti-inflammatory mediator EPA, and pronounced oxidative and inflammatory changes in the content of lipid mediators oxylipins. These changes include long-term elevation prostaglandins, which are synthesized from AA via COX-dependent pathways, as well as short bursts of LA derivatives that can be produced by both enzymatic and non-enzymatic free radical-dependent mechanisms. Premedication of the eyes using mitochondria-targeted antioxidant SkQ1 inhibited the upregulation of all oxylipins, whereas the accumulation of prostaglandins and lyso-PAF was specifically suppressed by topical treatment with COX inhibitor Nepafenac. Importantly, the most prominent antioxidant and anti-inflammatory benefits and overall retinal protective effects were achieved by the simultaneous administration of both drugs, indicating their synergistic action. Taken together, these novel findings add more insights to the mechanisms of antioxidant and anti-inflammatory action of Nepafenac and SkQ1, and they provide a rationale for their combined use in complex therapy of light maculopathy, AMD, and other retinal degenerative diseases.

## Abbreviations

AA	Arachidonic acid
AH	Aqueous humor
AMD	Age-related macular degeneration
BCA	Bicinchoninic acid
BV	Blood vessel
COX	Cyclooxygenases
CYP	Cytochrome P450 monooxygenases
DHA	Docosahexaenoic acids
9,10-DiHOME	9,10-dihydroxyoctadecamonoenoic acid
12,13-DiHOME	12,13-dihydroxyoctadecamonoenoic acid
EPA	Eicosapentaenoic acid
ERG	Electroretinography
9,10-EpOME	9,10-epoxyoctadecamonoenoic acid
12,13-EpOME	12,13-epoxyoctadecamonoenoic acid
GCL	Ganglion cell layer
9-HODE	9-hydroxyoctadecadienoic acid
13-HODE	13-hydroxyoctadecadienoic acid
INL	Inner nuclear retinal layer
IL-1	Interleukin-1
IL-10	Interleukin-10
IL-1β	Interleukin-1 beta
9-KODE	9-oxo-octadecadienoic acid
13-KODE	13-oxo-octadecadienoic acid
LTB4	Leukotriene B4
LIRD	Light-induced retinal degeneration
LA	Linoleic acid
LOX	Lipoxygenase
lyso-PAF	Lyso-Platelet-Activating Factor (1-O-hexadecyl-sn-glyceryl-3-phosphorylcholine)
MR	Medullary ray
NSAID	Nonsteroidal anti-inflammatory drug
ONL	Outer nuclear retinal layer;
OEA	Oleoylethanolamine ((Z)-N-(2-Hydroxyethyl)octadec-9-enamide)
Ph	Photoreceptor layer
PBS	Phosphate buffer saline
PAF	Platelet-activating factor
PUFA	Polyunsaturated fatty acid
PGD2	Prostaglandin D2
PGE2	Prostaglandin E2
PGF2α	Prostaglandin F2a
ROS	Reactive oxygen species
RPE	Retinal pigment epithelium
Sc	Sclera
SkQ1	10-(60-plastoquinonyl)-decyltriphenylphosphonium
TNF-α	Tumor necrosis factor alpha
UPLC-MS/MS	Ultra performance liquid chromatography-tandem mass spectrometry

## References

[1] Whitcup S.M., Nussenblatt R.B., Lightman S.L., Hollander D.A. (2013). Inflammation in retinal disease. Int. J. Inflamm..

[2] Campbell M., Humphries P. (2012). The blood-retina barrier: Tight junctions and barrier modulation. Adv. Exp. Med. Biol..

[3] Baksheeva V.E., Tiulina V.V., Tikhomirova N.K., Gancharova O.S., Komarov S.V., Philippov P.P., Zamyatnin A.A., Senin I.I., Zernii E.Y. (2018). Suppression of Light-Induced Oxidative Stress in the Retina by Mitochondria-Targeted Antioxidant. Antioxidants.

[4] Dammann O. (2010). Inflammation and retinopathy of prematurity. Acta Paediatr..

[5] Anderson D.H., Mullins R.F., Hageman G.S., Johnson L.V. (2002). A role for local inflammation in the formation of drusen in the aging eye. Am. J. Ophthalmol..

[6] Ebrahimi K.B., Handa J.T. (2011). Lipids, lipoproteins, and age-related macular degeneration. J. Lipids.

[7] Johnson M.W. (2009). Etiology and treatment of macular edema. Am. J. Ophthalmol..

[8] Hunter J.J., Morgan J.I., Merigan W.H., Sliney D.H., Sparrow J.R., Williams D.R. (2012). The susceptibility of the retina to photochemical damage from visible light. Prog. Retin. Eye Res..

[9] Zernii E.Y., Baksheeva V.E., Iomdina E.N., Averina O.A., Permyakov S.E., Philippov P.P., Zamyatnin A.A., Senin I.I. (2016). Rabbit Models of Ocular Diseases: New Relevance for Classical Approaches. CNS Neurol. Disord.-Drug Targ..

[10] Byrnes G.A., Chang B., Loose I., Miller S.A., Benson W.E. (1995). Prospective incidence of photic maculopathy after cataract surgery. Am. J. Ophthalmol..

[11] Baksheeva V.E., Gancharova O.S., Tiulina V.V., Iomdina E.N., Zamyatnin A.A., Philippov P.P., Zernii E.Y., Senin I.I. (2018). Iatrogenic Damage of Eye Tissues: Current Problems and Possible Solutions. Biochem. Biokhimiia.

[12] Wolffe M. (2016). How safe is the light during ophthalmic diagnosis and surgery. Eye.

[13] Wenzel A., Grimm C., Samardzija M., Reme C.E. (2005). Molecular mechanisms of light-induced photoreceptor apoptosis and neuroprotection for retinal degeneration. Prog. Retin. Eye Res..

[14] Zernii E.Y., Nazipova A.A., Gancharova O.S., Kazakov A.S., Serebryakova M.V., Zinchenko D.V., Tikhomirova N.K., Senin I.I., Philippov P.P., Permyakov E.A. (2015). Light-induced disulfide dimerization of recoverin under ex vivo and in vivo conditions. Free Radic. Biol. Med..

[15] Zernii E.Y., Nazipova A.A., Nemashkalova E.L., Kazakov A.S., Gancharova O.S., Serebryakova M.V., Tikhomirova N.K., Baksheeva V.E., Vladimirov V.I., Zinchenko D.V. (2019). Light-Induced Thiol Oxidation of Recoverin Affects Rhodopsin Desensitization. Front. Mol. Neurosci..

[16] Organisciak D.T., Vaughan D.K. (2010). Retinal light damage: Mechanisms and protection. Prog. Retin. Eye Res..

[17] Geiger P., Barben M., Grimm C., Samardzija M. (2015). Blue light-induced retinal lesions, intraretinal vascular leakage and edema formation in the all-cone mouse retina. Cell. Death Dis..

[18] Ghosh S., Shang P., Yazdankhah M., Bhutto I., Hose S., Montezuma S.R., Luo T., Chattopadhyay S., Qian J., Lutty G.A. (2017). Activating the AKT2-nuclear factor-kappaB-lipocalin-2 axis elicits an inflammatory response in age-related macular degeneration. J. Pathol..

[19] Zarbin M.A., Casaroli-Marano R.P., Rosenfeld P.J. (2014). Age-related macular degeneration: Clinical findings, histopathology and imaging techniques. Dev. Ophthalmol..

[20] Wooff Y., Man S.M., Aggio-Bruce R., Natoli R., Fernando N. (2019). IL-1 Family Members Mediate Cell Death, Inflammation and Angiogenesis in Retinal Degenerative Diseases. Front. Immunol..

[21] Mirshahi A., Hoehn R., Lorenz K., Kramann C., Baatz H. (2012). Anti-tumor necrosis factor alpha for retinal diseases: Current knowledge and future concepts. J. Ophthalmic Vis. Res..

[22] Ambati J., Atkinson J.P., Gelfand B.D. (2013). Immunology of age-related macular degeneration. Nat. Rev. Immunol..

[23] Deng Q., Wang Y., Wang C., Ji B., Cong R., Zhao L., Chen P., Zang X., Lu F., Han F. (2018). Dietary supplementation with omega-3 polyunsaturated fatty acid-rich oils protects against visible-light-induced retinal damage in vivo. Food Funct..

[24] Kim G.H., Paik S.S., Park Y.S., Kim H.G., Kim I.B. (2019). Amelioration of Mouse Retinal Degeneration After Blue LED Exposure by Glycyrrhizic Acid-Mediated Inhibition of Inflammation. Front. Cell. Neurosci..

[25] Toris C.B., Gulati V. (2011). The biology, pathology and therapeutic use of prostaglandins in the eye. Clin. Lipidol..

[26] Gabbs M., Leng S., Devassy J.G., Monirujjaman M., Aukema H.M. (2015). Advances in Our Understanding of Oxylipins Derived from Dietary PUFAs. Adv. Nutr..

[27] Schoenberger S.D., Kim S.J. (2013). Nonsteroidal anti-inflammatory drugs for retinal disease. Int. J. Inflamm..

[28] Bazan N.G., Reddy T.S., Bazan H.E., Birkle D.L. (1986). Metabolism of arachidonic and docosahexaenoic acids in the retina. Prog. Lipid Res..

[29] Tanito M., Yoshida Y., Kaidzu S., Ohira A., Niki E. (2006). Detection of lipid peroxidation in light-exposed mouse retina assessed by oxidative stress markers, total hydroxyoctadecadienoic acid and 8-iso-prostaglandin F-2 alpha. Neurosci. Lett..

[30] Morrow J.D., Awad J.A., Boss H.J., Blair I.A., Roberts L.J. (1992). Non-cyclooxygenase-derived prostanoids (F2-isoprostanes) are formed in situ on phospholipids. Proc. Natl. Acad. Sci. USA.

[31] Goel M., Picciani R.G., Lee R.K., Bhattacharya S.K. (2010). Aqueous humor dynamics: A review. Open Ophthalmol. J..

[32] Rubenstein D.A., Yin W., Frame M.D. (2015). Biofluid Mechanics: An Introduction to Fluid Mechanics, Macrocirculation, and Microcirculation.

[33] Mo J.S., Wang W., Kaplan H.J. (2007). Impact of inflammation on ocular immune privilege. Chem. Immunol. Allergy.

[34] Richer S.P., Rose R.C. (1998). Water soluble antioxidants in mammalian aqueous humor: Interaction with UV B and hydrogen peroxide. Vis. Res..

[35] Nucci C., Di Pierro D., Varesi C., Ciuffoletti E., Russo R., Gentile R., Cedrone C., Pinazo Duran M.D., Coletta M., Mancino R. (2013). Increased malondialdehyde concentration and reduced total antioxidant capacity in aqueous humor and blood samples from patients with glaucoma. Mol. Vis..

[36] McGahan M.C., Fleisher L.N. (1986). Antioxidant activity of aqueous and vitreous humor from the inflamed rabbit eye. Curr. Eye Res..

[37] Satici A., Guzey M., Gurler B., Vural H., Gurkan T. (2003). Malondialdehyde and antioxidant enzyme levels in the aqueous humor of rabbits in endotoxin-induced uveitis. Eur. J. Ophthalmol..

[38] Beyazyildiz E., Cankaya A.B., Beyazyildiz O., Ergan E., Celik H.T., Yilmazbas P., Ozturk F. (2014). Disturbed oxidant/antioxidant balance in aqueous humour of patients with exfoliation syndrome. Jpn. J. Ophthalmol..

[39] Terao N., Koizumi H., Kojima K., Yamagishi T., Yamamoto Y., Yoshii K., Kitazawa K., Hiraga A., Toda M., Kinoshita S. (2018). Distinct Aqueous Humour Cytokine Profiles of Patients with Pachychoroid Neovasculopathy and Neovascular Age-related Macular Degeneration. Sci. Rep..

[40] Feng S.F., Yu H.H., Yu Y., Geng Y., Li D.L., Yang C., Lv Q.J., Lu L., Liu T., Li G.D. (2018). Levels of Inflammatory Cytokines IL-1 beta, IL-6, IL-8, IL-17A, and TNF-alpha in Aqueous Humour of Patients with Diabetic Retinopathy. J. Diabetes Res..

[41] Hillier R.J., Ojaimi E., Wong D.T., Mak M.Y.K., Berger A.R., Kohly R.P., Kertes P.J., Forooghian F., Boyd S.R., Eng K. (2017). Aqueous Humor Cytokine Levels as Biomarkers of Disease Severity in Diabetic Macular Edema. Retin. J. Ret. Vit. Dis..

[42] Takai Y., Tanito M., Ohira A. (2012). Multiplex Cytokine Analysis of Aqueous Humor in Eyes with Primary Open-Angle Glaucoma, Exfoliation Glaucoma, and Cataract. Investig. Ophthalmol. Vis. Sci..

[43] Novikova Y.P., Gancharova O.S., Eichler O.V., Philippov P.P., Grigoryan E.N. (2014). Preventive and therapeutic effects of SkQ1-containing Visomitin eye drops against light-induced retinal degeneration. Biochemistry.

[44] Ke T.L., Graff G., Spellman J.M., Yanni J.M. (2000). Nepafenac, a unique nonsteroidal prodrug with potential utility in the treatment of trauma-induced ocular inflammation: II. In vitro bioactivation and permeation of external ocular barriers. Inflammation.

[45] Kauppinen A., Paterno J.J., Blasiak J., Salminen A., Kaarniranta K. (2016). Inflammation and its role in age-related macular degeneration. Cell. Mol. Life Sci. CMLS.

[46] Sennlaub F., Auvynet C., Calippe B., Lavalette S., Poupel L., Hu S.J., Dominguez E., Camelo S., Levy O., Guyon E. (2013). CCR2(+) monocytes infiltrate atrophic lesions in age-related macular disease and mediate photoreceptor degeneration in experimental subretinal inflammation in Cx3cr1 deficient mice. EMBO Mol. Med..

[47] Yorston D. (2006). What’s new in age-related macular degeneration?. Community Eye Health.

[48] El Baba F., Jarrett W.H., Harbin T.S., Fine S.L., Michels R.G., Schachat A.P., Green W.R. (1986). Massive hemorrhage complicating age-related macular degeneration. Clinicopathologic correlation and role of anticoagulants. Ophthalmology.

[49] Spindler J., Zandi S., Pfister I.B., Gerhardt C., Garweg J.G. (2018). Cytokine profiles in the aqueous humor and serum of patients with dry and treated wet age-related macular degeneration. PLoS ONE.

[50] Jaffe G.J., Dick A.D., Brezin A.P., Nguyen Q.D., Thorne J.E., Kestelyn P., Barisani-Asenbauer T., Franco P., Heiligenhaus A., Scales D. (2016). Adalimumab in Patients with Active Noninfectious Uveitis. New Engl. J. Med..

[51] Sacca S.C., Roszkowska A.M., Izzotti A. (2013). Environmental light and endogenous antioxidants as the main determinants of non-cancer ocular diseases. Mutat. Res..

[52] Dolz-Marco R., Gallego-Pinazo R., Pinazo-Duran M.D., Pons-Vazquez S., Domingo-Pedro J.C., Diaz-Llopis M. (2014). Intravitreal docosahexaenoic acid in a rabbit model: Preclinical safety assessment. PLoS ONE.

[53] Tanito M., Anderson R.E. (2009). Dual roles of polyunsaturated fatty acids in retinal physiology and pathophysiology associated with retinal degeneration. Clin. Lipidol..

[54] Thivierge M., Rola-Pleszczynski M. (1995). Up-regulation of inducible cyclooxygenase gene expression by platelet-activating factor in activated rat alveolar macrophages. J. Immunol..

[55] Elison J.R., Weinstein J.E., Sheets K.G., Regan C.E., Lentz J.J., Reinoso M., Gordon W.C., Bazan N.G. (2018). Platelet-Activating Factor (PAF) Receptor Antagonism Modulates Inflammatory Signaling in Experimental Uveitis. Curr. Eye Res..

[56] Rosenbaum J.T., Angell E., Wilson D., Broquet C., Boney R.S., Braquet P. (1999). Intravitreally injected platelet activating factor induces retinitis in experimental animals. Curr. Eye Res..

[57] Secchi A.G., Fregona I., D’Ermo F. (1979). Lysophosphatidyl choline in the aqueous humour during ocular inflammation. Br. J. Ophthalmol..

[58] Eakins K.E., Whitelocke R.A., Perkins E.S., Bennett A., Unger W.G. (1972). Release of prostaglandins in ocular inflammation in the rabbit. Nat. New Biol..

[59] Miller J.D., Eakins K.E., Atwal M. (1973). The release of PGE2-like activity into aqueous humor after paracentesis and its prevention by aspirin. Investig. Ophthalmol..

[60] Unger W.G., Perkins E.S., Bass M.S. (1974). The response of the rabbit eye to laser irradiation of the iris. Exp. Eye Res..

[61] Csukas S., Paterson C.A., Brown K., Bhattacherjee P. (1990). Time course of rabbit ocular inflammatory response and mediator release after intravitreal endotoxin. Investig. Ophthalmol. Vis. Sci..

[62] Chen E., Benz M.S., Fish R.H., Brown D.M., Wong T.P., Kim R.Y., Major J.C. (2010). Use of nepafenac (Nevanac) in combination with intravitreal anti-VEGF agents in the treatment of recalcitrant exudative macular degeneration requiring monthly injections. Clin. Ophthalmol..

[63] Acar U., Acar D.E., Tanriverdi C., Acar M., Ozdemir O., Erikci A., Ornek F. (2017). Prostaglandin E2 Levels of Aqueous and Vitreous Humor in Ketorolac 0.4% and Nepafenac 0.1% Administered Healthy Rabbits. Ocul. Immunol. Inflamm..

[64] Yoshida Y., Umeno A., Shichiri M. (2013). Lipid peroxidation biomarkers for evaluating oxidative stress and assessing antioxidant capacity in vivo. J. Clin. Biochem. Nutr..

[65] Masuda T., Shimazawa M., Hara H. (2017). Retinal Diseases Associated with Oxidative Stress and the Effects of a Free Radical Scavenger (Edaravone). Oxid. Med. Cell. Longev..

[66] Bian M.J., Du X.Y., Cui J.G., Wang P.W., Wang W.J., Zhu W.L., Zhang T., Chen Y. (2016). Celastrol protects mouse retinas from bright light-induced degeneration through inhibition of oxidative stress and inflammation. J. Neuroinflamm..

[67] Kubota S., Kurihara T., Ebinuma M., Kubota M., Yuki K., Sasaki M., Noda K., Ozawa Y., Oike Y., Ishida S. (2010). Resveratrol Prevents Light-Induced Retinal Degeneration via Suppressing Activator Protein-1 Activation. Am. J. Pathol..

[68] Zernii E.Y., Golovastova M.O., Baksheeva V.E., Kabanova E.I., Ishutina I.E., Gancharova O.S., Gusev A.E., Savchenko M.S., Loboda A.P., Sotnikova L.F. (2016). Alterations in tear biochemistry associated with postanesthetic chronic dry eye syndrome. Biochemistry.

[69] Zernii E.Y., Gancharova O.S., Baksheeva V.E., Golovastova M.O., Kabanova E.I., Savchenko M.S., Tiulina V.V., Sotnikova L.F., Zamyatnin A.A., Philippov P.P. (2017). Mitochondria-Targeted Antioxidant SkQ1 Prevents Anesthesia-Induced Dry Eye Syndrome. Oxid. Med. Cell. Longev..

[70] Zernii E.Y., Gancharova O.S., Tiulina V.V., Zamyatnin A.A., Philippov P.P., Baksheeva V.E., Senin I.I. (2018). Mitochondria-targeted antioxidant SKQ1 protects cornea from oxidative damage induced by ultraviolet irradiation and mechanical injury. BMC Ophthalmol..

[71] Chistyakov D.V., Grabeklis S., Goriainov S.V., Chistyakov V.V., Sergeeva M.G., Reiser G. (2018). Astrocytes synthesize primary and cyclopentenone prostaglandins that are negative regulators of their proliferation. Biochem. Biophys. Res. Commun..

